# The Built Environment and Health: Introducing Individual Space-Time Behavior

**DOI:** 10.3390/ijerph6061724

**Published:** 2009-05-26

**Authors:** Dick Saarloos, Jae-Eun Kim, Harry Timmermans

**Affiliations:** 1 Centre for the Built Environment and Health, School of Population Health, The University of Western Australia, M707, 35 Stirling Highway, Crawley WA 6009, Australia; E-Mail: dick.saarloos@uwa.edu.au; 2 Institute of Island Culture, Mokpo National University, 61 Dorim-Ri, Cheonggye-Myeon, Muan-Gun, Jeonnam 534-729, Korea; E-Mail: ecokimje@yahoo.co.kr; 3 Urban Planning Group, Department of Architecture, Building and Planning, Eindhoven University of Technology, P.O. Box 513, 5600 MB Eindhoven, The Netherlands

**Keywords:** built environment, overweight and obesity, individual-based modeling, space-time behavior, activity patterns, health impact assessment

## Abstract

Many studies have examined the relationship between the built environment and health. Yet, the question of how and why the environment influences health behavior remains largely unexplored. As health promotion interventions work through the individuals in a targeted population, an explicit understanding of individual behavior is required to formulate and evaluate intervention strategies. Bringing in concepts from various fields, this paper proposes the use of an activity-based modeling approach for understanding and predicting, from the bottom up, how individuals interact with their environment and each other in space and time, and how their behaviors aggregate to population-level health outcomes.

## 1. Introduction

Human behavior depends on the environment in which it takes place, while in turn people also influence the environment by their presence and activities [1,2]. As part of the environment, people behave in response to both physical and social settings [3]. In fact, every individual not only adapts to his or her physical and social environment but also makes up part of the social environment of other individuals [4]. Obviously, there are more forces affecting both individuals and the environment. For instance, businesses and organizations located in the environment will influence where people travel, while governments and institutions exercise rules and mechanisms that affect the behavior of individuals, households, businesses and organizations. An understanding of this multi-layered network of interactions is required when environmental design is intended as an instrument to establish desired behavior, e.g., to encourage walking and cycling, or to create safer places.

Over the last two decades, the field of health promotion has demonstrated increased attention for the possible impact of the environment on health. It has been recognized that the channels of impact are multi-faceted and exist at multiple levels of hierarchy. A social-ecological perspective [5] has become the common approach to categorize the various environmental influences on individual health behavior. Besides the roles of individual and interpersonal characteristics, a social-ecological approach distinguishes the impacts of organizational and institutional circumstances, as well as aspects related to the community and society [6]. As such, it is a comprehensive framework to identify the various levels at which interventions are possible, such as person-oriented and environment-oriented strategies. It also highlights the need of multidisciplinary approaches that integrate perspectives and methods from health promotion and other sectors. However, despite the consensus about the legitimacy of an holistic view, epidemiological research is still strongly oriented at isolating the effects of particular attributes rather than using more systematic and integrative approaches that could map out causal pathways between interventions and health outcomes [7]. This leaves a gap between research, and policy and practice. The quality of decision-making regarding intervention strategies would greatly improve if the focus were on understanding the many intertwined cause-effect relationships and when this would be translated in ways readily useable in policy and practice. The challenge is how to effectively align public health and urban planning, and to optimally integrate the strengths of both these fields, to enable creating healthy and sustainable environments [8].

Numerous studies have appeared in the literature providing evidence of the association between physical activity and the environment in which people live [9]. Initially, these studies focused on only individual determinants, but the field has progressed towards social-ecological studies acknowledging that behavior is influenced by multi-level factors of various origins [10] and across multiple levels of scale [11]. Although limited by being cross-sectional evidence only, the findings give reason to believe that interventions to change environments by removing barriers and providing more opportunities for physical activity can be effective [12]. While current studies fulfill a primary task by identifying correlates of physical activity or advancing methods to objectively measure the environment, for research on interventions to progress systematically the mechanisms of action must be studied [13]. The science, however, is not sufficiently advanced [9]. The current line of research is not answering the question how the various factors of influence work together in terms of system dynamics [14]. Nor is it addressing how and why the environment is actually influencing behavior; the focus is only on *outcomes* of behavior and these are linked to at best crude proxies representing features of the built environment [15]. In current practice some interventions may prove effective but, in the absence of more sophisticated models that could predict the effects of interventions on actual human behavior, decision-making is more haphazard than it should.

This paper aims to encourage discussion among researchers and practitioners in the fields of health promotion and public health about the need to address the individual space-time behavior that underlies the interaction between the built environment and health. Focused on overweight and obesity, the paper will bring in concepts from the fields of behavioral geography, ecology, and land use and transportation planning to propose an activity-based modeling approach to systematically address the complexities involved in the relationship between the built environment and health. A discussion is included in which the ideas brought forward will be merged, addressing how resultant models should be envisioned, what issues will require attention for implementation, and how such models can assist in bridging the gap between research and practice.

## 2. Environmental Influences on Overweight and Obesity

In the last decade, rapidly increasing rates of overweight and obesity have become a major public health concern around the world. For instance, in Europe, the prevalence of overweight (i.e., a body mass index between 25 and 29.9 kg/m^2^) now ranges between 32–79% in men and 28–78% in women, while the prevalence of obesity (i.e., a body mass index of 30 kg/m^2^ or more) ranges between 5–23% in men and 7–36% in women [16]. A strong evidence base exist about the various health consequences of overweight and obesity, such as cardiovascular problems, type 2 diabetes, certain cancers and psychosocial problems [17]. Many epidemiological studies have been dedicated to identifying those segments of the population in which overweight or obesity is most prevalent. Generally, it is found that those who are female, middle-aged, ethnic minority, unemployed or in unskilled jobs, lower income, less educated, living with others, married, parents, rural, and/or living in particular regions are more likely to be obese [18]. This wide range of factors reveals some of the complexity underlying the overweight and obesity issue.

In search for the causes, it is widely agreed upon that the increasing rates have occurred too rapidly to be primarily due to genetic factors and, thus, that changes in non-genetic factors must be playing a major role [19]. Overweight and obesity strongly depend on behavioral issues. Simply stated, weight gain results when energy consumption exceeds energy expenditure for a prolonged period [19]. Hence, nutrition and physical activity behaviors can be identified as key factors. Somehow people have gradually changed their eating habits and turned to more sedentary lifestyles. While interdependencies between the two behaviors may exist, several contextual factors seem to have contributed to the rise of overweight and obesity. The fast food industry has been able to flourish in our modern society driven by consumption, convenience and marketing. At the same time, technological changes, such as workplace automation and the dominance of the car for personal travel, have substantially reduced the physical activity that was naturally embedded in daily life [9]. Many more of such factors have gradually changed the environment in which people live, work and play.

With the majority of the population living in cities, it is sensible to look at how urban environments have changed, influenced lifestyles, and contributed to the increase in overweight and obesity. Urban sprawl is widely criticized for its negative impacts on public health [20]. The widespread implementation of car-oriented land use and transportation developments has systematically ignored or underestimated the underlying public health consequences [21]. Sprawl has resulted in monotonous neighborhoods with low land-use diversity, low residential density, low employment density, and low street connectivity. In these environments, there is heavy reliance on car use and people spend more time on travelling instead of on health-improving activities [21]. This leaves them less time with family or friends and less time to devote to community activities, making it more difficult to develop and sustain supportive social relationships [8,20].

Several reviews on environmental factors influencing overweight and obesity already exist [22–24]. These all indicate that most attention has been given to links between the built environment and physical activity [9,25]. In the context of residential environments, the following built environment characteristics are normally associated with lower rates of overweight and obesity [16]:

- Higher density, mixed land use and street connectivity;
- More opportunities for active transport (walking, cycling);
- Better access to public transportation;
- Better access to facilities for physical activity and open spaces;
- Higher road safety and safety from crime; and
- More aesthetically pleasing street design.

Environmental influences on nutrition behavior have received less attention until more recently [26,27]. Use of the term ‘food environment’ has become customary in this respect. Although sometimes including influences from media/information, the term usually refers to the available opportunities in an area to obtain food [28]. As such, the food environment can be seen as a thematic subset of the built environment that includes food stores (supermarkets, grocery stores) and restaurants (fast food and full-service restaurants). Due to site selection and other marketing principles applied by food stores and restaurants, it is clear that there is not an even spatial distribution of healthy food choices within cities [29]. Typically, studies apply ‘access to food’ measures, such as the distance to the nearest food store or restaurant, or the density of such facilities within a defined area [23]. Most studies are limited to making associations between the presence and proximity of food stores or restaurants within a geographic area and a set of socio-economic characteristics of the population within that area [30,31]. Besides accessibility, also affordability of food is considered to be related to obesity [23,32].

Because the built environment is inhabited and used by individuals, it coexists with a social environment that can foster (or undermine) social connectivity and capital, facilitating (or hampering) interactions with others [33,34]. A rapidly growing evidence base indicates that, across the life course, the structure and quality of social interactions have profound effects on psychological, behavioral and physiological functioning, health and wellbeing [35]. There is widespread agreement that both physical and social environmental factors can encourage or discourage people to make health-conscious choices related to their behavior [10,36].

Obviously, individuals differ in their behaviors. Besides socio-demographic characteristics, physical activity and nutrition behaviors are considered to be affected by an array of psychological, cognitive, or emotional-level attributes of a person, including psychological health, attitudes, beliefs, motivations, preferences and self-efficacy [37,38]. Such aspects will determine a person’s perception of opportunities and constraints in the environment. In association with obesity, it has been found that there are differences between perceived and observed measures of the environment [39]. Although the built, social and food environment can all be objectively measured, the literature suggests that people’s perception of the environment might be more closely related to their actual behavior [40].

Much remains to be learned about the relative importance of the individual, the social context and the physical environment as determinants of health behaviors [9,37]. Findings often remain inconclusive as size and direction of effects tend to vary between studies [40], pointing to the fact that many of the complexities underlying the relationship between the built environment and health behaviors are poorly understood.

## 3. Complexities

Understanding the causal pathways and mechanisms between an intervention and behavioral outcomes is important if the full ramifications of policies are to be understood [41]. However, many questions still remain to be answered. Especially, the conceptualization of the built environment is not agreed upon [9], and the heterogeneity of human behavior is not well understood.

Studies investigating the relationship between the built environment and health behaviors have usually investigated the effects of only a limited number of built environment characteristics, and mainly with a restricted focus on suburban areas. They have used only crude proxies for the relevant geographic areas and for the attributes that may be important [15,37]. It is not clear how individuals perceive the neighborhood space and scale, and how they filter spatial information when making their behavioral choices [42]. Proper delineation of the area of influence – i.e., ‘the neighborhood’ – is an important prerequisite for correct assessment of environmental impacts on health [43–45]. Often, studies assume neighborhoods to be formed by the artificial boundaries of census districts, simply because of data-related convenience [23,46]. In recent years, however, there is a growing use of individual-based neighborhoods that capture the area of influence around each person’s home. Still, further detail is needed to account for differences between population segments, especially based on age [23,47]. Moreover, even a single individual’s neighborhood can be ‘fluid’ and vary depending on time, place or issue [34,44].

The identification of variables and measurements to reliably capture the influence of the built environment is also found to be complicated. Multicollinearity between attributes has led to the common use of factors or composite indices which are often difficult to translate to policies and interventions [48]. Consideration of neighborhood attributes has most importantly materialized into a construct known as *walkability* [46,49], a composite measure that – simply stated – expresses the suitability of an area for walking. Walkability usually includes aspects of land-use mix, street connectivity (or intersection density) and residential density of neighborhoods. Studies aim at finding associations between the walkability of neighborhoods and, for instance, different types and levels of physical activity or the prevalence of overweight and obesity. Increasingly, studies also include the *local opportunities* for physical activity or food consumption, i.e., potential destinations that are available within a walkable distance. Most attention has been given to shops (e.g., food stores) and public open spaces, while using measures such as the number of destinations or the distance to the nearest destination. Commonly, these measures ignore qualitative aspects of destinations (e.g., safety of a park or its layout) that will affect their attractiveness. Overall, there are many facets that differentiate neighborhoods. The choice of variables, however, is often driven by data availability. Theoretical frameworks to conceptualize and operationalize environmental factors and their relationships in a health behavior context are rare. The Behavioral Model of Environment [3,40] is a framework that distinguishes three spatial constructs promoting walking: origins and destinations of trips, routes connecting origins and destinations, and areas around origins and destinations. Although many variables have been used to measure the built environment [3], normally they are only based on assumptions about features being relevant for certain population segments.

There is a lack of broader elaborations about the multiple and complex ways in which the built environment may influence behavior for diverse populations and diverse settings [38]. Cities are complex communities of heterogeneous individuals, and understanding how the urban context affects the health of inhabitants requires consideration of multiple, often competing, influences [50]. The relationship between the built environment and health behaviors operates through many mediating factors, such as socio-demographic characteristics, personal and cultural variables, safety and security issues, time allocation, travel-related and environmental attitudes, and perceptions regarding built environment attributes [9,42]. Such factors can have a direct influence on behavior or indirectly by modifying the sensitivity to built environment characteristics [42]. Moreover, the same environment will have different meaning to different people, influencing them in different ways [2]. The relative importance of built environment features and the mechanisms through which they influence people’s behavior are likely to vary for different segments of the population, such as for men and women, for families compared with elderly or children, and for persons or groups with different social, economic or cultural background [23,40,47,51]. In addition, it is even possible that different attributes have different spatial extents of influence on behavioral choices of different individuals [42]. Variation in geographic scale of impacts will, in part, also be due to differences in mobility. For instance, someone without a car may be impacted by the food environment within walking distance, while for car owners the food environment could encompass a much larger area. Furthermore, variability is likely to exist in relation to the purpose of activity, such as walking for recreation or walking for transport [9], and the type of environment. Obesity, physical inactivity and associated diseases appear in both suburban and inner-city areas but as a result of processes that seem different [34].

A typical flaw in research investigating the impact of the built environment on health behaviors is that it overlooks the fact that neighborhoods do not exist in isolation [34,52]. The local neighborhood is nested in larger and more complex environments, and interdependencies exist among immediate and more distant environments [5]. Moreover, because people travel, they are exposed to many different environments [23]. The ease with which they can travel to destinations in the wider environment is likely to affect the use of facilities in the local neighborhood and the choice to walk. This relates not only to space issues but also to time, which is an aspect that has not been well accounted for in examining the relationship between the built environment and health behaviors [9].

To improve health behaviors it is widely agreed upon that a combination of intervention approaches will be necessary [53]. Changes to the built environment should be accompanied by person-oriented interventions aimed at improving persons’ attitude, self-efficacy, perception, and so on [54]. However, the exact mechanisms through which combined interventions can have synergetic effects are still largely unknown. To better target interventions, further investigations are required to understand how interventions affect people’s behavior while taking notice of their diversity, and which interventions will be most effective for a neighborhood given the characteristics of its residents.

## 4. Methodological Shift

Several academics [7,8,55,56] have suggested the use of system thinking, mainly based on the concepts of the social-ecological framework, to ensure a comprehensive look at the factors influencing health behaviors at different hierarchical levels. However, the field has not reached further than a few attempts to map or conceptualize the interrelationships and relative importance of various factors [57,58]. The system of mechanisms underlying health behaviors in their social-ecological settings refers to human group processes that are highly complex, nonlinear, path dependent, self-organizing and dynamic [59,60]. To understand the operation of such systems, a modeling approach is needed that is different from the common statistical models that try to capture system regularities under restrictive or unrealistic assumptions including linearity, homogeneity, normality, and stationarity [61,62].

In reality, individuals are the actual building blocks of the system under study. The aggregate properties of interest in a population or community (e.g., the prevalence of overweight and obesity) emerge from the behaviors of individuals in response to their environment and each other. Instead of trying to model this at the system-level, it appears more natural and fruitful to apply a bottom-up approach called *individual-based modeling* (also known more generally as agent-based modeling) [4]. This approach has taken root in the field of ecology that, similar to public health, is concerned with populations and communities. Historically, ecological modelers also applied system-level models [63], capturing ecosystems by highly aggregated analytical models that at best reduce behavior to a few differential equations. Given the complexities faced in ecological studies, nowadays an individual-based modeling approach is considered to have important advantages to understand and predict ecosystems. As opposed to the traditional approach, it can naturally deal with the fact that individuals change in many ways over their life course by adapting to their environmental circumstances [4]. Also, it can take account of the fact that, since individuals differ from each other, their interactions with the environment and each other will be largely unique. Furthermore, feedback loops in which the emergent structure influences individuals can easily be incorporated. For instance, if an intervention would succeed in having residents spend more time on outdoor activities in their neighborhood, this may improve aspects like social cohesion and sense of safety, as a result of which residents may further increase their outdoor activities.

It is important to note that individual-based modeling requires a fundamentally different mindset in terms of understanding and thinking about a system. It implies directly modeling individuals, their behaviors, and their interactions, in order to observe, manipulate and understand the behavior of the whole system [64]. System-level properties (e.g., physical activity levels in a community) only *emerge* as a result of adaptive individuals, all with their own behavioral rules, interacting with each other and with their environment (e.g., residents being influenced by family or friends, by seeing others being physically active, or by having recreational facilities within a walkable distance). Hence, an individual-based approach requires, not only observing population-level properties in different environments, but also studying the processes by which behavior of individuals are affected by the physical and social aspects of an environment, how individuals adapt and how their interactions lead to system-level properties.

**Figure 1.**
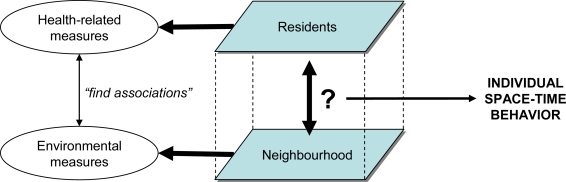
The contemporary approach to study environmental impacts on health behaviors (left), and the missing link of individual space-time behavior (right).

While the overall objective should be to understand health behaviors of people in response to their environment, there is a dearth of attempts to develop models that capture adaptive individual traits to predict behavior under varying circumstances. Typically, studies measure several characteristics of a neighborhood by means of GIS routines that often show great similarity to landscape metrics widely used in the field of landscape ecology [65]. In addition, non-spatial data are collected from residents about their physical activity (intensity, duration and frequency), nutrition (type, amount and frequency) or weight status. Next, regression-based techniques are applied to identify associations between the environmental measures and these health-related measures (Figure 1). This approach sidesteps how health behaviors actually occur as a result of individuals interacting with their environment and with each other [52,62]. To model and predict environmental impacts on health behaviors such as physical activity and nutrition, it will be necessary to understand how different activities are linked in space and time at the individual level [66]. More specifically, it needs to be understood which health-related activities people are conducting where, when, for how long, with whom and so on.

## 5. Space-Time Behavior

Spatial behavior in response to the built environment is a topic that can be assigned to the field of behavioral geography. Since the 1970s, this field has produced theories and models of preference and choice that are widely used to predict the likely impacts of policy decisions on spatial behavior and to assess the feasibility of envisaged projects [67]. Different types of individual behavioral choice models have been developed that explicitly relate choice behavior to the environment through the consideration of perceptions, preference formation and decision making. Individuals are assumed to develop a cognitive representation of the environment, which gives them an impression of the available alternatives (or ‘choice set’) for conducting a certain activity or trip. By judging several attributes, they are assumed to add a certain utility to each alternative. This results in a preference structure that positions the alternatives in terms of overall utility or preference. The eventual choice is assumed to be systematically related to this structure. As people learn from the choices they make, however, their perceptions, preference formation and decision making can change over time.

From this description it is clear that analyzing individual space-time behavior will offer several challenges. Both individuals and environments will demonstrate a large degree of variability. The available choice sets will differ between persons, they will evaluate the alternatives based on different sets of attributes, and they will make different trade-offs when deciding. Furthermore, all these aspects can vary over space and time. Since the 1990s, the field of land use and transportation planning has seen a rapid increase in so-called *activity-based modeling* (see next section), which conceptualizes activity patterns of individuals as sequences of activities over space and time, governed by opportunities and a variety of constraints [68]. Some research has been emphasizing particular facets of space-time behavior (e.g., activity duration, time allocation, trip-chaining and stop-pattern formation), but there is an increasing number of studies suggesting more comprehensive models of activity-travel patterns [69]. For instance, models now often contain elements of institutional context and also address intra-household decision making.

Determining the choice set available to an individual requires careful consideration [70]. As mentioned earlier, it is reasonable to say that behavior in the local neighborhood will be influenced by the wider environment [34,52]. In terms of choice behavior, whenever a person considers conducting an out-of-home activity, knowing facilities located outside the neighborhood means that this person will have alternatives to local facilities. As for location, the relative attractiveness (or utility) of such alternatives may increase when they are located near a person’s work location, along the route to/from work, or spatially clustered with other types of useful facilities. Even in the simplest case of a single-purpose trip starting from home, most persons are likely to have multiple alternatives to choose from. For example, when needing groceries, walking to a local store is an option that might be chosen because of health considerations. Thinking of the possibly heavy bags to bring back home, taking the car to visit that same store might be preferred because of convenience. Still, when knowing a more distant store with a larger product variety or lower prices, a trip to that store may even be more attractive or rewarding. Likewise, in the case of deciding to go walking or jogging, the choice outcome may not necessarily be the local street network or park if one could take the car and drive to a more distant park with better facilities or more attractive scenery.

Based on principles of time geography [71] it is possible to identify the ‘action space’ of individuals [72], which is a space-time measure of individual accessibility. For determining a person’s choice set and predicting his or her activity pattern, the *potential* action space is important. This can be defined as the area containing all activity locations that a person could reach, subject to a set of temporal and spatial constraints, such as the distance between bases (e.g., the person’s home and fixed work location), available time intervals between activities, and travel-time ratios [73]. Similar but with a focus on actual behavior, a ‘physical activity space’ (PAS) measure has recently been suggested to map where an individual spends time and engages in physical activity [74]. When attuned to capturing the *potential* physical activity space, such a measure would identify the opportunities in space and time an individual will have for physical activity during a day, be it in the local neighborhood or not. Whether opportunities will actually be used will depend on the value an individual adds to different types of activities and on his or her time budget. Basically, the available time budget will depend on an individual’s daily activity pattern, and his or her trade-offs between different activities. These aspects are likely to vary between weekdays and weekends.

So-called stated preference and choice experiments [75] are well-established techniques to elicit the attributes that people assess when evaluating choice alternatives. Such experiments use a systematic design process in which attributes and their levels are predefined and varied to create sets of hypothetical alternatives from which respondents are asked to choose or indicate their preference. This has several advantages over ‘revealed’ experiments (i.e., experiments based on actual behavior), as it will enable avoiding effects of confounders and allow unbiased estimation of attribute effects. Outcomes of choice experiments can, for instance, shed light on the preferences of older people for attributes of local parks [76]. In addition, operations research tools and methods such as decision trees and influence diagrams [77] can be used to derive people’s decision rules and heuristics from activity diary data [78,79].

In summary, to understand the spatial and temporal dimensions of how a neighborhood could influence health behaviors among its residents, it seems warranted to broaden the view beyond the boundaries of the neighborhood, to examine how health-enhancing activities fit into larger activity patterns, and to study aspects of the underlying individual choice behavior. Data are required that will let us analyze current patterns of daily activities inside and outside the local neighborhood to see how attractive local facilities are to non-local ones, and to assess which attributes of choice alternatives are relevant to whom. The next section will give a brief introduction to activity-based modeling, which is the mainstream approach used in land use and transportation modeling to forecast travel demand. This approach is believed to provide us key concepts of individual space-time behavior needed for thinking about and investigating environmental impacts on health behaviors.

## 6. Activity-Based Modeling

Activity-based models simulate which activities people are conducting, where, when, for how long, with whom, the transport mode used and possibly some other characteristics. The level of representation of time and space tends to be high, implying that very detailed simulations and predictions are possible. The activity-based approach goes back to various disciplines. It was originally suggested in urban planning and geography but especially in mid 1990 it received a highly significant impulse in transportation research, with several hundred of publications annually. Most of this output concerns the results of empirical analyses of particular aspects of activity-travel patterns, but in addition several operational models have become available in recent years. New technologies have led to or promise new advances in data collection, giving new opportunities for the further development of activity-based models.

In the mid 1990s, transportation researchers advocated to replace so-called four-step models by activity-based models. The four-step approach simulated travel by predicting, at an aggregate level, trip generation, destination choice, mode choice and route choice as independent processes. Activity-based models have been advocated for a variety of reasons. First, empirical evidence suggests that an increasingly larger percentage of trips are not home-based as a result of changing distributions of land use, time pressure and higher car availability rates. Because traditional transport demand models are based on home-based trips, they introduce systematic error and the amount of error has significantly increased.

Secondly, the policy agenda has changed. Whereas traditionally transport demand forecasting models were developed to assess the feasibility and impact of major new infrastructure, since the 1990s this policy orientation has been supplemented with a focus on transport demand management. In addition, environment-related concerns have been added to the policy agenda. These changing policies have triggered a need for a more detailed modeling system, increasing resolution in both time and space (more detailed time period than peak/off-peak and from traffic zones to postal codes or even geo-coded information).

Thirdly, empirical research has shown that response patterns to transport demand management can be quite diverse. It led to the need of modeling interdependencies between choice facets of transport demand as opposed to the assumed independence of trip-based models. Another reason for developing activity-based models is to improve the consistency of travel forecasting by developing a more integrated approach. For example, speed on the transportation network dictates the action spaces of travelers. Similarly, job departure times in the afternoon will be correlated with home departure times in the morning. Likewise, leaving the car at home for the work commute may create more options for other members of the household and therefore influence their activity-travel patterns.

Examples are a study by Vovsha and colleagues [80] reporting the development and application of discrete choice based models in the United States (Portland, New York City, San Francisco County and Columbus, Ohio), CEMDEP [81] applied in Texas, FAMOS [82] and its predecessor PCATS that have found application in Japan and Florida, and ALBATROSS [68,83] that is currently used by the Dutch Ministry of Transport. Although not applied in the same sense, TASHA [84,85] is now also ready for application. A more detailed discussion is provided in [69].

Advances in model development were made possible by new developments in data collection. Activity-based models require fine-grained details about human activities. Therefore, activity diaries or time use diaries have been dominantly used to collect the data required for the estimation of activity-based models. These instruments record the sequence of activities conducted during a full day and corresponding information on transport mode, start and end times, travel party, and destination. The organizing principle underlying activity-travel diaries is the sequence of activities. In case of time use diaries, the information is collected by asking about successive time intervals.

The land use and transportation literature has been concerned with transport choices rather than with physical activity, most often from the viewpoint of utilitarian travel. Although activity diaries and time-use surveys have allowed collection of rich data on travel behavior, they normally do not provide adequate estimates of physical activity [86]. Two general trends in data collection for activity-based analysis and modeling can be distinguished that will ease this problem. First, keeping in line with traditional travel surveys, *active* data collection on activity-travel patterns has been further developed. Active data collection means that respondents are asked directly to provide information about the various facets of their activity-travel patterns. Because diary data require more time and are more prone to errors, there has been a tendency to replace or supplement paper-and-pencil questionnaires with (Internet-based) computer-assisted or electronic survey instruments. Second, much research has recently been devoted to examine the possibilities of *passive* data collection. This means that modern technology such as GPS, (GPS enabled) cellular phones, RFID chips, etc. are used to trace respondents. Data fusion and data mining algorithms are then used to derive data about other facets of activity-travel behavior from such records. These new data collection techniques are likely to improve the geographical detail and accuracy of data, making activity-based models suitable for addressing health behaviors in small-scale areas such as local neighborhoods. It will permit to look beyond simple availability and untested measures of accessibility, yielding knowledge on the actual use of specific resources within a community as well as on the frequency, duration, and sequencing of activities in space and time [87].

## 7. Discussion

**Figure 2.**
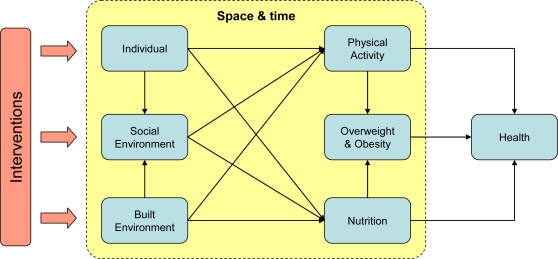
Space-time behavior as the underlying concept for studying the health impacts of environmental interventions (here related to overweight and obesity).

Public health interventions are aimed at establishing health-enhancing changes at the population (or system) level. Any intervention, however, will have to work through the individuals that make up the population. Hence, choosing the most effective intervention strategy requires an explicit understanding of how individuals will respond. It is the task of academic researchers to build up a comprehensive knowledge base of this individual behavior to inform and influence policy and practice. Given the documented impacts of the built environment on health behaviors, various interventions can be considered to influence the way people behave in response to the environment in which they live, work and play (Figure 2). These include actual changes to the built environment (e.g., providing more opportunities for recreation, improving access to healthy food, and creating places to accommodate social events for community members), interventions that address individuals (e.g., campaigns aimed at improving attitudes to physical activity, nutrition and social interactions, or increasing awareness of the health benefits or risks involved), and interventions to encourage social activities (e.g., community programs to promote physical activity and healthy food consumption). To support decision-making about health promotion interventions, studies will need to aim at understanding and predicting how such interventions will change individual health behaviors in space and time, and how this aggregates to population-level health outcomes (Figure 2).

Although space-time behavior is a largely unexplored area for researchers in health promotion and public health, it is the central focus in land use and transportation planning. This field has seen an evolution from aggregate models to disaggregate models. Now, activity-based modeling is the state-of-the-art approach to model and predict individual space-time behavior. Albeit that this approach is mostly finding application at the level of cities or regions, its general concepts can easily be applied to smaller areas. This is especially so, now that the adoption of new data collection techniques such as GPS instruments is giving access to data at the fine-grained scale needed for studies at the local neighborhood level. Adoption of this methodology could materialize the highly needed move from the general thesis that neighborhoods affect health to the specifics of how and why this may occur [15]. Oriented at individual behavior, it offers an integrated perspective in which the focus is not on statistical relationships between characteristics of the built environment and particular health issues, but on how the built environment affects, among other factors, the way people organize their activities and travel in space and time and how this in turn affects their health.

**Figure 3.**
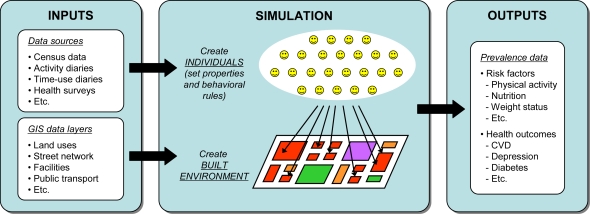
Schematic overview of an activity-based model of health behavior.

In general terms, an activity-based model of health behavior would allow examining and predicting which health-related activities people conduct where, when, for how long, and with whom. This would enable us to explicitly investigate many of the causal pathways between the built environment and health that are assumed to exist by the current line of research. An activity-based model of health behavior should be envisioned as an operational instrument that simulates the activities of individuals to forecast the likely impacts of interventions on health outcomes and risk factors at the population-level (Figure 3). Simulation is often seen as the only practical way to study complex systems and, thus, the most suitable instrument to test the feasibility of intended decisions or to predict their impacts [88]. The individual-based nature of the approach will address issues in close resemblance to how they occur in reality by letting population-level properties emerge from the behavior of individuals. Hence, model development implies modeling individuals as computational entities with autonomous and heterogeneous abilities of perception, intelligence and action [89]. To develop realistic individual-based models, several aspects require attention [63]:

- *Emergence*: What behaviors and population dynamics should emerge from the model? How should individual traits be modeled so that realistic population responses emerge?
- *Adaptation*: Given the model’s temporal and spatial scales, what adaptive processes of individuals should be modeled? Which mechanisms do individuals use to adapt in response to which environmental forces?
- *Fitness and strategy*: What objectives and strategies are appropriate to use as the basis for modeling individual decision making?
- *State-based responses*: How should decision processes depend on an individual’s state?
- *Prediction*: What are realistic assumptions about how individuals anticipate the consequences of decisions?

Because models are developed from the viewpoint of individuals, the approach has the advantage of being well-suited for incremental development of models aimed at simulating a complex system. Initially, one can set up the overall structure by creating (i) a virtual representation of a built environment and (ii) a virtual population of individuals with some behavioral rules to make activity choices while interacting with the environment and with each other [90]. Further development will involve enhancing or adding behavioral rules to capture a certain desired effect. When the representation of behavior has reached a satisfactory level, the approach will provide great flexibility to test models in different or increasingly complex environments. This would only require bringing the model’s population in line with the population of the environment to be investigated.

Individual-based models can operate as virtual laboratories to run controlled experiments of hypothetical interventions [62,88,91]. Through simulation, they allow a comprehensive and structured assessment of impacts of different interventions on health behaviors in a population under study. This gives them potential as an instrument for prospective quantitative Health Impact Assessment (HIA) [41,92,93]. HIA addresses the possible effects of a policy, program or project on the health of a population and the distribution of those effects within the population [94]. Individual-based models will be naturally suitable to account for individual differences in exposure and susceptibility to environmental conditions dependent on personal, geographical or temporal characteristics. This will enable obtaining refined estimates of health impacts and to see how various groups in the population respond to an intervention [41].

Given a user interface that allows one to understand the model, interact with it and interpret its outcomes [63], an activity-based model of health behavior can serve different purposes and have value to different parties involved in health promotion, public health and urban planning. First, by enabling experimentation with interventions it can be an instrument for researchers to develop models of increasing sophistication and to investigate possible synergies between interventions. As such, it can also serve a role in education [66]. Second, an activity-based model of health behavior can operate as a tool to facilitate communication between academics (or modelers) and policy-makers, practitioners or planners. By simulating how interventions operate through mechanisms of individual behavior to eventually result in outcomes for a population, models can be designed to visualize how outcomes come about. Such insight can give directions in decision-making and play a role in advocating the most suitable and effective interventions for a given case. Finally, visualization can also play a facilitating role in public participation [95] to reach consensus about interventions when changes to the built environment are suggested to community members.
